# *Pholcusmaxian* sp. nov., the fifth endemic spider species of *Pholcusphungiformes* species-group (Araneae: Pholcidae) at the border between Jilin, China and North Korea

**DOI:** 10.3897/BDJ.9.e72464

**Published:** 2021-09-16

**Authors:** Ying Lu, Fan Yang, Qiaoqiao He

**Affiliations:** 1 College of Life Science, Shenyang Normal University, Shenyang, China College of Life Science, Shenyang Normal University Shenyang China

**Keywords:** taxonomy, morphology, biodiversity, new species, Northeast Asia

## Abstract

**Background:**

The distribution pattern of *Pholcusphungiformes* species-group indicates that, in Jilin and Heilongjiang, China and North Korea, additional species diversity remains undiscovered.

**New information:**

*Pholcusmaxian* sp. nov., one new species of *P.phungiformes* species-group, is described, based on material collected from the borderline between Jilin, China and North Korea. It represents the fifth endemic species of this species-group and the genus *Pholcus* from this region.

## Introduction

**Figure 1.**
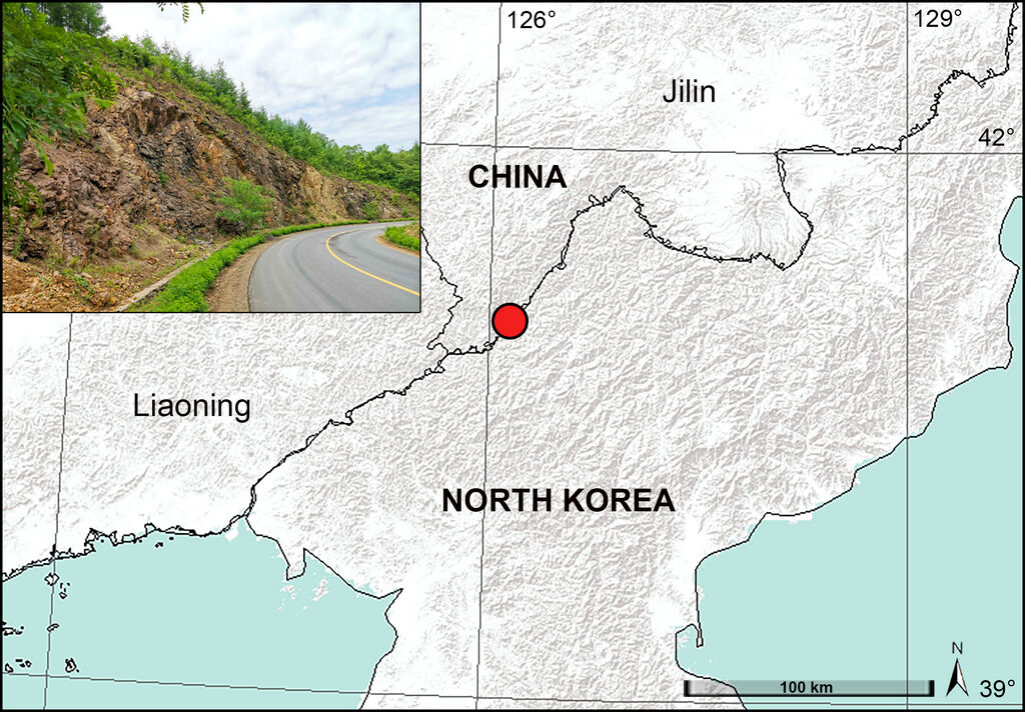
Distribution record and habitat of *Pholcusmaxian* sp. nov. from the border between Northeast China and North Korea.

The genus *Pholcus* Walckenaer, 1805 belongs to the family Pholcidae C.L. Koch, 1850, is diverse and currently contains 95 genera and 1,842 species (World Spider Catalog 2021). *Pholcus* is mainly distributed in the Palaearctic, Indo-Malayan, Afrotropical and Australasian Regions, and includes 21 species-groups (Huber 2011). One of the generic species-groups, the *P.phungiformes* species-group exhibits high species diversity and currently contains 80 species (Wang et al. 2020, Yao et al. 2021, Lee et al. 2021). These species mainly occur on rock walls in three large mountain ranges: the Taihang Mountains in southern North China (n = 22 spp.), the Changbai Mountains at the border between Northeast China and North Korea (n = 25 spp., of which 20 are in Liaoning, four in Jilin, one in Heilongjiang, China and 0 in North Korea) and the Taebaek Mountains on the Korean Peninsula (n = 32 spp., of which 31 are in South Korea, one in North Korea). Additionally, only *P.phungiformes* Oliger, 1983 occurs further east, probably as a result of human transport (Huber 2011). This distribution pattern indicates that additional species diversity in Jilin and Heilongjiang, China and North Korea remains undiscovered. In the present study, we describe a new species assigned to the *P.phungiformes* species-group, collected at the border between Jilin, China and North Korea (Fig. 1). This new species represents the fifth endemic species of the *P.phungiformes* species-group and the genus *Pholucs* from this region (Previously, there existed only four endemic species: *P.lingulatus*, *P.sublingulatus*, *P.wangjiang* and *P.yuhuangshan*).

## Materials and methods

Specimens were examined and measured with a Leica M205 C stereomicroscope. The left male pedipalp was photographed. External female genitalia were photographed before the dissection. Vulva was previously treated in a 10% warm solution of potassium hydroxide (KOH) to dissolve soft tissues before illustration. Images were captured with a Canon EOS 750D wide zoom digital camera (24.2 megapixels) mounted on the stereomicroscope mentioned above and assembled using Helicon Focus 3.10.3 image stacking software (Khmelik et al. 2005). All measurements are given in millimetres (mm). Leg measurements are shown as: total length (femur + patella + tibia + metatarsus + tarsus). Leg podomeres were measured on their dorsal side. The distribution map was generated with ArcGIS 10.2 (Esri Inc.). The specimens studied are preserved in 75% ethanol and deposited in the College of Life Science, Shenyang Normal University (SYNU) in Liaoning, China. Terminology and taxonomic descriptions follow Huber (2011) and Yao et al. (2015). The following abbreviations are used in the descriptions: ALE = anterior lateral eye, AME = anterior median eye, PME = posterior median eye, L/d = length/diameter; used in the illustrations: b = bulbal, da = distal apophysis, e = embolus, fa = frontal apophysis, pa = proximo-lateral apophysis, pp = pore plate, pr = procursus, u = uncus.

## Taxon treatments

### Pholcus maxian sp. n.

816556D7-FC86-5173-A04F-89780DCF1A36

8A2D135A-502A-4377-862E-8877912805C3

#### Materials

- **Type status:**Holotype. **Occurrence:** recordedBy: Zhiyuan Yao; individualCount: 1; sex: male; lifeStage: adult; **Taxon:** order: Araneae; family: Pholcidae; genus: Pholcus; **Location:** country: China; stateProvince: Jilin; municipality: Tonghua, Ji’an; locality: Maxian Town; verbatimLocality: Shanghuolong Village, roadside of G331; verbatimElevation: 213 m a.s.l.; verbatimLatitude: 41°4.43'N; verbatimLongitude: 126°6.41'E; **Event:** samplingProtocol: by hand; year: 2020; month: 6; day: 27; **Record Level:** institutionCode: SYNU-Ar00141
- **Type status:**Paratype. **Occurrence:** recordedBy: Zhiyuan Yao; individualCount: 3; sex: 1 male, 2 females; lifeStage: 3 adults; **Taxon:** order: Araneae; family: Pholcidae; genus: Pholcus; **Location:** country: China; stateProvince: Jilin; municipality: Tonghua, Ji’an; locality: Maxian Town; verbatimLocality: Shanghuolong Village, roadside of G331; verbatimElevation: 213 m a.s.l.; verbatimLatitude: 41°4.43'N,; verbatimLongitude: 126°6.41'E; **Event:** samplingProtocol: by hand; year: 2020; month: 6; day: 27; **Record Level:** institutionCode: SYNU-Ar00142–00144

#### Description

**Figure 2.**
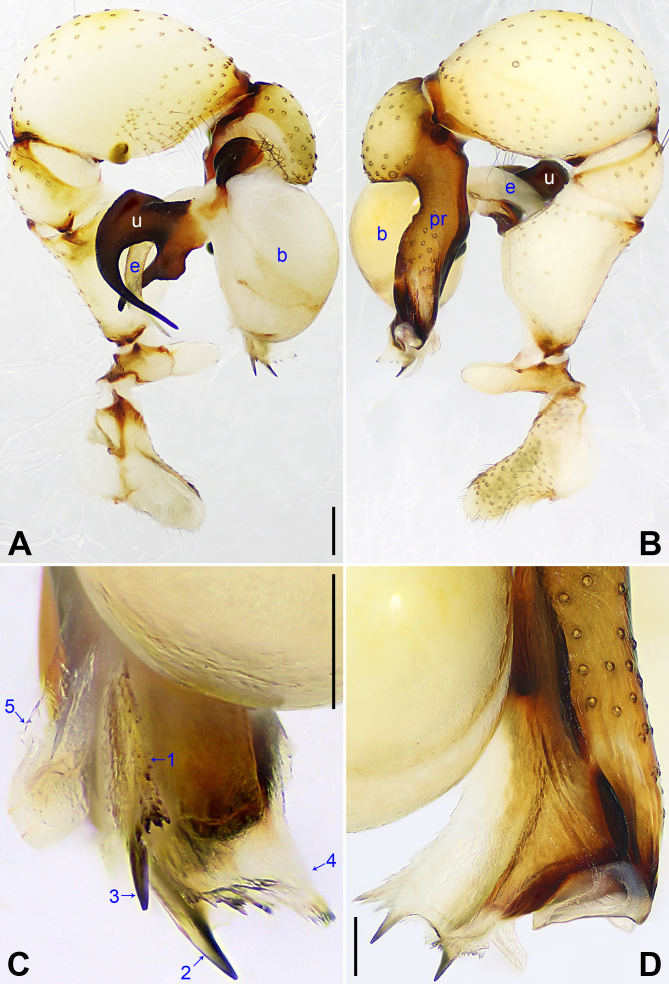
*Pholcusmaxian***sp. nov.**, holotype male **A** Pedipalp, prolateral view; **B** Pedipalp, retrolateral view; **C** Distal part of procursus, prolateral view, arrows 1–5 point at curved, prolatero-distal membranous process, two pointed sclerotised apophyses, prolatero-dorsal membranous lamella and prolatero-ventral membranous process, respectively; **D** Distal part of procursus, dorsal view. b = bulb, e = embolus, pr = procursus, u = uncus. Scale bars: 0.20 (A–B), 0.10 (C–D).

**Figure 3.**
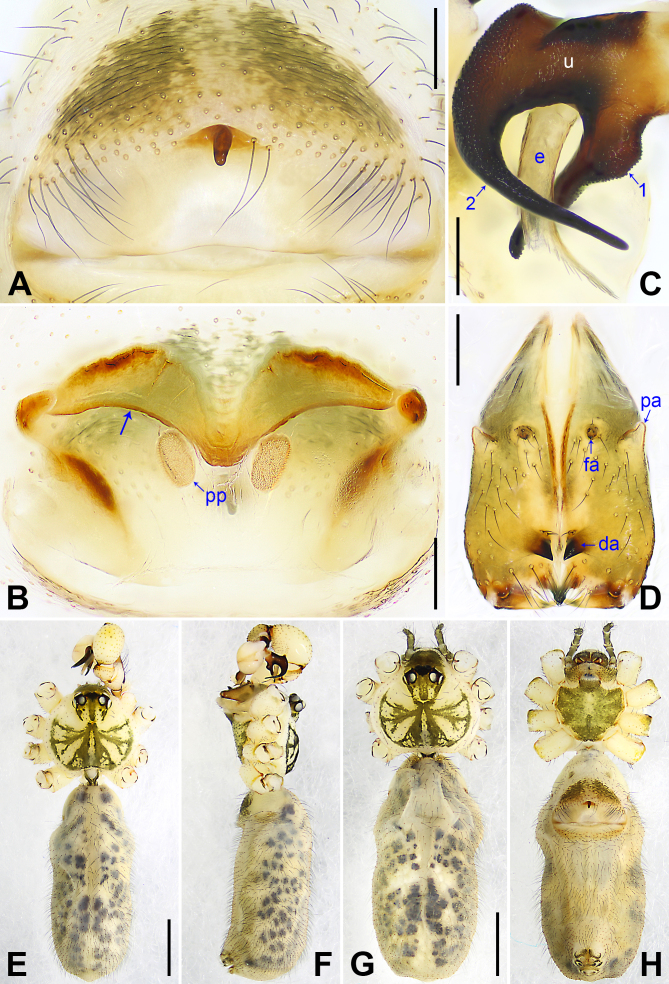
*Pholcusmaxian***sp. nov. A** Paratype female, external genitalia, ventral view; **B** Paratype female, vulva, dorsal view, arrow points at posterior part of arch; **C** Holotype male, bulbal apophyses, prolateral view, arrows 1–2 point at proximal and distal apophysis; **D** Holotype male, chelicerae, frontal view; **E** Holotype male, habitus, dorsal view; **F** Holotype male, habitus, lateral view; **G** Paratype female, habitus, dorsal view; **H** Paratype female, habitus, ventral view. da = distal apophysis, e = embolus, fa = frontal apophysis, pa = proximo-lateral apophysis, pp = pore plate, u = uncus. Scale bars: 0.20 (A–D), 1.00 (E–H).

**Male** (holotype, Figs 2, 3): Total length 5.10 (5.24 with clypeus), carapace 1.24 long, 1.65 wide, opisthosoma 3.86 long, 1.50 wide. Leg I: 38.76 (9.78 + 0.78 + 10.25 + 15.62 + 2.33), leg II: 26.84 (7.58 + 0.67 + 6.76 + 10.22 + 1.61), leg III: 19.20 (5.61 + 0.65 + 4.62 + 7.11 + 1.21), leg IV: 26.09 (7.79 + 0.52 + 6.79 + 9.52 + 1.47); tibia I L/d: 60. Distance PME-PME 0.24, diameter PME 0.14, distance PME-ALE 0.04, distance AME-AME 0.06, diameter AME 0.11. Sternum wider than long (1.05/0.93). Habitus as in Fig. 3E–F. Carapace yellowish, with brown radiating marks and marginal brown band; ocular area yellowish, with median and lateral brown bands; clypeus brown; sternum yellowish, with brown marks. Legs yellowish, but dark brown on patellae and whitish on distal parts of femora and tibiae, with darker rings on subdistal parts of femora and proximal and subdistal parts of tibiae. Opisthosoma yellowish, with dorsal and lateral spots. Ocular area elevated, without eye stalks. Thoracic furrow absent. Chelicerae (Fig. 3D) with pair of proximo-lateral apophyses, pair of distal apophyses with two teeth each and pair of frontal apophyses. Pedipalps as in Fig. 2A–B; trochanter with long (much longer than wide), retrolaterally strongly bulged ventral apophysis; femur with retrolatero-proximal apophysis and indistinct ventral protuberance; tibia with prolatero-ventral projection; procursus simple proximally, but complex distally, with curved prolatero-distal membranous process (arrowed 1 in Fig. 2C) with two pointed, sclerotised apophyses (arrowed 2–3 in Fig. 2C), flat prolatero-dorsal membranous lamella (arrowed 4 in Fig. 2C) and prolatero-ventral membranous process (arrowed 5 in Fig. 2C); uncus with long, proximally swollen and distally slender proximal apophysis (arrowed 1 in Fig. 3C), slender, strongly curved distal apophysis (arrowed 2 in Fig. 3C) and scales; appendix absent; embolus weakly sclerotised, with some transparent distal projections (Fig. 3C). Retrolateral trichobothrium of tibia I at 2% proximally; legs with short vertical setae on tibiae, metatarsi and tarsi, without spines or curved setae; tarsus I with 33 distinct pseudosegments.

**Female** (Fig. 3): Similar to male, habitus as in Fig. 3G–H. Total length 5.08 (5.22 with clypeus), carapace 1.46 long, 1.71 wide, opisthosoma 3.62 long, 1.72 wide; tibia I: 7.44; tibia I L/d: 41. Distance PME-PME 0.21, diameter PME 0.15, distance PME-ALE 0.04, distance AME-AME 0.05, diameter AME 0.11. Sternum wider than long (1.07/0.92). External female genitalia (Fig. 3A) simple and flat, with n-shaped median mark and knob. Vulva (Fig. 3B) with pair of wing-like, sclerotised anterior arch and pair of elliptic pore plates.

**Variation**: Tibia I in another paratype male (SYNU-Ar00142): 11.16. Tibia I in another paratype female (SYNU-Ar00144): 7.89.

#### Diagnosis

The species resembles *P.hamatus* Tong & Ji, 2010 (see Tong and Ji 2010: figs. 1a–c, j, 2a–g; Yao et al. 2021: figs. 2B.7, S9A–D) with similar male chelicerae (Fig. 3D) and external female genitalia (Fig. 3A), but can be distinguished by procursus with large, prolatero-dorsal membranous lamella (arrowed 4 in Fig. 2C; very small in *P.hamatus*) and with prolatero-ventral membranous process (arrowed 5 in Fig. 2C; absent in *P.hamatus*), by uncus with long, proximally swollen and distally slender proximal apophysis (arrowed 1 in Fig. 3C; proximal apophysis short and swollen in *P.hamatus*) and by vulval anterior arch strongly curved posteriorly (arrowed in Fig. 3B, arch eyebrow-shaped; posteriorly slightly curved (arch nearly trapezoidal) in *P.hamatus*).

#### Etymology

The specific name refers to the type locality and is a noun in apposition.

#### Distribution

China (Jilin, type locality; Fig. 1).

#### Biology

The species was found on rock walls.

## Supplementary Material

XML Treatment for Pholcus maxian
